# Maribavir in lung transplant recipients with cytomegalovirus infection: real-life experience

**DOI:** 10.1007/s15010-026-02927-3

**Published:** 2026-08-18

**Authors:** Tobias Veit, Stefan Trost, Teresa Kauke, Nina Engels, Gabriela Leuschner, Michael Gerckens, Carlo Mümmler, Sebastian Michel, Michael Zoller, Valeria Hackemann, Jürgen Behr, Nikolaus Kneidinger

**Affiliations:** 1 Department of Medicine V, LMU University Hospital, Comprehensive Pneumology Center (CPC-M), LMU Munich, Member of the German Center for Lung Research (DZL), Marchioninistrasse 15, 81377 Munich, Germany; 2 Laboratory for Immunogenetics, University of Munich, LMU, Munich, Germany; 3 Division of Thoracic Surgery, LMU University Hospital, LMU Munich, Member of the German Center for Lung Research (DZL), Munich, Germany; 4 Department of Cardiac Surgery, LMU University Hospital, LMU Munich, Member of the German Center for Lung Research (DZL), Munich, Germany; 5 Department of Anaesthesiology, University Hospital, LMU, Munich, Germany; 6 Max Von Pettenkofer Institute and Gene Center, Virology, National Reference Center for Retroviruses, LMU, Munich, Germany; 7 Department of Internal Medicine, Division of Respiratory Medicine, Lung Research Cluster, Medical University of Graz, Auenbruggerplatz 15, 8036 Graz, Austria; 8 Institute of Lung Health and Immunity (LHI), Comprehensive Pneumology Center (CPC), Helmholtz Munich, Member of the German Center of Lung Research (DZL), Munich, Germany

**Keywords:** Solid organ transplantation, Lung transplantation, CMV, Maribavir, Refractory/resistant

## Abstract

**Introduction:**

Cytomegalovirus remains a major challenge in LTx recipients. Despite advances in antiviral therapy, refractory and resistant CMV infections persist, and real-world data on maribavir, a recently approved UL97 kinase inhibitor, are limited.

**Methods:**

We retrospectively analyzed 49 LTx recipients treated with maribavir for refractory and resistant (R/R) CMV between August 2022 and December 2024.

**Results:**

Overall, 34 patients (69.4%) achieved a sustained virological response, defined as complete CMV DNA clearance, with a > 1 log₁₀ viral load decline after a median of 11.5 days and complete viral clearance after a median of 24.8 [7–54] days. Sixteen patients (32.7%) failed therapy; UL97 resistance mutations were detected during maribavir therapy in 14 patients (28.6%). Non-responders had higher baseline viral loads (> 10,000 IU/ml: 53.3% vs. 20.6%; p = 0.047) and slower viral decline (14 (6–85) vs. 10.5 (2–27) days; p = 0.033). In multivariable analysis, higher baseline CMV viral load was independently associated with maribavir resistance (OR 3.729 per log₁₀ increase, 95% CI 1.360–10.226; p = 0.011).

**Conclusion:**

In conclusion, maribavir represents an important therapeutic option for R/R CMV after lung transplantation. However, treatment failure, relapse, and resistance remained common, highlighting the need for careful monitoring and further optimization of treatment strategies.

**Supplementary Information:**

The online version contains supplementary material available at https://doi.org/10.1007/s15010-026-02927-3.

## Introduction

Cytomegalovirus (CMV) infection is one of the most significant opportunistic infections in allogeneic hematopoietic stem cell transplant (HSCT) and solid organ transplant (SOT) recipients, including lung transplant (LTx) patients, and is associated with increased morbidity and mortality [1, 2]. It can lead to graft dysfunction, rejection, and other serious complications [3, 4]. Managing CMV infections, particularly those that are resistant or refractory to standard antiviral therapies, presents a significant challenge. Standard antiviral drugs like ganciclovir, valganciclovir, foscarnet, and cidofovir are limited by their toxicities, including myelosuppression and nephrotoxicity, which complicates long-term use [4].

Maribavir, a benzimidazole riboside, represents a novel therapeutic option with a distinct mechanism of action. It inhibits the UL97 protein kinase, which is essential for CMV replication, and has shown activity against CMV strains resistant to other antivirals [5]. Phase 2 and 3 clinical trials have highlighted maribavir’s favorable safety profile and efficacy, particularly in patients with R/R CMV infections. These trials indicate successful outcomes with maribavir, even in cases where other therapies have failed [6, 7].

Although several real-world studies have reported the effectiveness of maribavir in mixed solid organ and hematopoietic transplant populations, data specifically addressing lung transplant recipients remain scarce. A recent multicenter French study by Kamar et al. demonstrated virological clearance in approximately half of transplant recipients and a low incidence of treatment-emergent resistance, while Daher et al. reported successful CMV control in the majority of patients in a smaller real-world cohort [8, 9]. Further research regarding best treatment and prophylaxis of R/R CMV infection is needed to reflect best clinical practice choices. However, understanding the optimal duration of antiviral therapy is critical, as premature discontinuation may lead to viral relapse, while prolonged treatment increases the risk of resistance development [10, 11].

The introduction of new antiviral agents such as maribavir and letermovir, which is approved for CMV prophylaxis in allogenic hematopoietic stem cell transplant recipients, has the potential to transform CMV management [6, 12–16]. However, both drugs pose a low genetic barrier to resistance, emphasizing the importance of monitoring resistance development [4, 17]. Currently, data on the rates and risk factors associated with maribavir resistance in LTx recipients are rare. Given the high burden of CMV infection in this population, understanding the clinical risk factors for resistance is crucial for optimizing antiviral strategies.

This study aims to provide a comprehensive analysis of real-life data on the use of maribavir in lung transplant recipients at a large transplant center. By examining the efficacy, safety, and duration of treatment with maribavir, this study seeks to offer insights into optimizing CMV management in this vulnerable population and address the gap in existing knowledge regarding long-term outcomes and resistance development in LTx patients.

## Methods

### Study cohort

This was a single-center, retrospective cohort study of lung transplant recipients treated with maribavir for refractory (with or without resistance) CMV infection and/or disease between August 2022 and December 2024. The study was conducted at the University of Munich, Germany, and was approved by the local ethics committee (UE No. 19–060). The requirement for informed consent was waived by the ethics committee due to the retrospective nature of the study. Eligible patients had undergone lung transplantation at our center between February 2017 and December 2024. During this transplantation period, a total of 611 lung transplantations were performed at our center, providing the source population from which the study cohort was identified.

Refractory CMV infection was defined as failure to achieve a significant log_10_ decrease (i.e. < 1 log10) in CMV DNA after 14 days or more of appropriately dosed antiviral treatment [18]. Rescue therapy with maribavir was also initiated in case of resistant CMV infection, defined as one or more genetic mutation associated with resistance to ganciclovir/valganciclovir, foscarnet, cidofovir and / or letermovir, if the refractory criteria were met. Before the approval of maribavir in Europe (November 2022), a cost coverage for off-label use was applied and approved by the patient’s respective health insurance company prior to treatment initiation. Maribavir treatment was applied as described for treatment of refractory CMV-infection and disease in adult HSCT and SOT recipients (400 mg orally twice daily) [6]. Each patient was followed up for a minimum of four months after initiation of maribavir.

Treatment response was defined as ≥ 1 log₁₀ reduction in CMV viral load followed by complete CMV viral clearance within 8 weeks after initiation of maribavir. Non-responder (treatment failure) was defined as follows: i) persistent viral load after 4 weeks of antiviral therapy without significant log10 decrease (i.e., < 1 log10), ii) missing CMV viremia clearance at the end of week 8 or iii) increase of viral load (i.e., ≥ 1 log10) over the course of treatment. Viral clearance was defined as a plasma CMV DNA level below the lower limit of quantification of the assay (< 32 IU/mL). Thus, both samples with undetectable CMV DNA and samples reported as detected but not quantifiable (< 32 IU/mL) were considered to have achieved viral clearance. Viral clearance had to be confirmed in two consecutive plasma samples obtained at least 3 days apart.

### CMV monitoring

Before transplantation, fully automated serological tests for anti-CMV immunoglobulin IgG were conducted on both the recipient and the donor. After transplantation CMV-polymerase chain reaction (PCR) was assessed twice weekly during hospital stay. Upon discharge, CMV-PCR and tacrolimus trough levels were monitored weekly during the first two years after lung transplantation. Thereafter, lifelong CMV-PCR monitoring was continued, with the interval gradually extended from every two weeks to every three weeks and finally to a maximum of every four weeks, depending on the patient’s clinical status and CMV risk profile. Throughout follow-up, all CMV-PCR results were assessed at our centre by a transplant physician. Patients were routinely followed at our center through both outpatient clinic visits and inpatient assessments. Scheduled outpatient follow-up visits were performed at least every three months during the first two years after transplantation, with additional unscheduled outpatient visits or inpatient assessments whenever clinically indicated.

CMV DNA was quantified using the cobas® CMV Test (Roche Diagnostics), a quantitative real-time PCR assay performed on EDTA plasma. The assay had a lower limit of detection of 35 copies/mL (approximately 32 IU/mL) [16]. Specifically, the CMV-specific cell-mediated immunity was assessed by use of T-Track CMV (Lophius Biosciences GmbH, Regensburg, Germany) and performed according to manufacturer’s instructions on request of the treating physician. The assay was performed to evaluate the recovery of CMV-specific immune responses and to support individualized clinical decision-making regarding antiviral treatment duration and the likelihood of virological control. CMV-specific cell-mediated immunity was not assessed at predefined time points. Whenever feasible, testing was preferentially performed before initiation and/or before discontinuation of antiviral therapy. However, as the assay is not part of routine clinical diagnostics at our institution and requires separate sample processing, these time points could not be consistently implemented. A test was considered positive if the IFN-γ response to CMV peptides results in > 10 spots IE-1 and/or > 10 spots pp-65. CMV end-organ disease was assessed in clinically indicated tissue biopsies and body fluid specimens. Proven CMV disease was defined by histopathological evidence of CMV-associated cytopathic changes and positive CMV immunohistochemistry. Detection of CMV DNA in cerebrospinal fluid in the absence of clinically relevant blood contamination, together with compatible clinical manifestations of central nervous system disease, was considered diagnostic of CMV central nervous system disease. Blood contamination was assessed based on routine cerebrospinal fluid analysis and clinical interpretation rather than a predefined cerebrospinal fluid red blood cell threshold [4].

### Antiviral prophylaxis and treatment

Maintenance immunosuppression after lung transplantation consisted of mycophenolate mofetil, prednisone, and a calcineurin inhibitor (tacrolimus or ciclosporin A). Induction therapy was not routinely administered but was reserved for selected patients, including those undergoing retransplantation or those with preformed donor-specific HLA antibodies. According to the pre-transplant CMV donor/recipient serostatus, valganciclovir prophylaxis (900 mg once daily, adjusted for renal function) was administered for 9–12 months in high-risk recipients (D + /R −), 6 months in intermediate-risk recipients (D-/R + ; D + /R +), and 3 months in low-risk recipients (D − /R −). In the case of significant increase in CMV-PCR upon, the prophylactic dose was increased to a therapeutic dose (900 mg every 12 h and based on creatinine clearance, respectively). There was no predefined CMV DNA threshold for initiation of treatment-dose valganciclovir. In routine clinical practice, therapeutic dosing was generally initiated in patients with detectable, clinically relevant CMV DNAemia confirmed by at least two consecutive positive CMV-PCR measurements. The final decision to initiate treatment was made at the discretion of the treating transplant physician based on the CMV viral load and the individual patient’s risk profile. In patients with treatment failure, either admission to our hospital with initiation of intravenous ganciclovir (5 mg/kg body weight every 12 h, adjusted for creatinine clearance) and intravenous CMV immune globulin (CMVIgG; Cytotect®) at a dose of 100 IE/kg/day (equivalent to 1 ml/kg/day or 50 mg/kg/day) for 5–7 days was performed, or, in selected cases, rescue therapy with letermovir was initiated as previously described [16]. No adjustment of the prednisolone dose was performed for CMV infection. In all patients with CMV infection and/or disease, maintenance immunosuppression was adjusted according to the individual clinical situation. This included reduction or temporary discontinuation of mycophenolate mofetil (MMF) and reduction of target through level of tacrolimus and/or switch of the standard immunosuppressive regimen to a mammalian target of rapamycin (mTOR)-inhibitor-based quadruple low tacrolimus immunosuppression regimen, with a target trough level of 3–5 ng/mL for both drugs [16]. The decision to initiate maribavir was made by the treating transplant physician on an individual clinical basis. Maribavir was used as rescue therapy in patients with refractory CMV infection or disease after failure of one or more prior standard antiviral therapies and/or in the presence of genotypically confirmed antiviral resistance. In cases of persistent refractory CMV disease, CMV-specific T-cell therapy was administered. Clinical-grade CMV-specific T-cell products were obtained through the alloCELL network (Hannover Medical School, Germany). T cells were generated under GMP-compliant conditions using the IFN-γ CliniMACS Cytokine Capture System (Miltenyi Biotec). Donors were selected from the original stem cell donor, a haploidentical family donor, or a partially HLA-matched unrelated third-party donor from the alloCELL registry [19]. Leukocytopenia was defined as a leukocyte blood count < 3.5 G/L, determined by fluorescence flow cytometry. Neutropenia was defined as absolute neutrophil count (ANC) < 1500 G/L.

### Genotype testing

Genotypic testing for antiviral resistance was routinely performed at our institution whenever antiviral resistance was clinically suspected and resistance testing was requested by the treating transplant physician. Genotypic testing for ganciclovir, foscarnet, cidofovir, letermovir, and maribavir resistance was performed by PCR amplification followed by Sanger sequencing of the relevant regions of the UL97 (amino acids 312–666), UL54 (amino acids 265–1053), UL56 (amino acids 12–393), UL51, and UL89 genes. Sanger sequencing was performed by Eurofins Genomics (Ebersberg, Germany), whereas sequence analysis and interpretation of resistance-associated mutations were carried out at the Max von Pettenkofer Institute and Gene Center, LMU Munich. Phenotypic characterization, i.e. assessment of ganciclovir concentration required for 50% viral growth inhibition (50% effective concentration [EC_50_]) to assess the degree of resistance and therapeutic dose adjustment according to the level of the resistance was not performed.

### Statistical analysis

Continuous variables are presented as mean ± standard deviation (SD) or median [Q1, Q3], as appropriate, whereas categorical variables are summarized as frequencies and percentages. Univariate analyses were performed using the Mann–Whitney U test for continuous variables. Categorical variables were compared using Pearson’s chi-square test or Fisher’s exact test, as appropriate. As multiple statistical comparisons were performed without adjustment for multiple testing, the reported p-values should be interpreted as exploratory and hypothesis-generating. Multivariable logistic regression analysis was performed to identify variables independently associated with the detection of maribavir resistance. Variables for the regression model were selected based on statistical significance in the univariate analysis (p < 0.05) while considering the risk of overfitting and multicollinearity. Baseline CMV viral load was entered as log10(IU/mL). Results are reported as odds ratios (ORs) with 95% confidence intervals (CIs) and p-values. A two-sided p-value < 0.05 was considered statistically significant. Statistical analyses were performed using IBM SPSS Statistics, version 24.0 (IBM Corp., Armonk, NY, USA).

## Results

### Study population/profile of maribavir-treated patients

During the study period, a total of 49 lung transplant recipients received rescue therapy with maribavir for R/R CMV infection and were assessed for treatment response. At baseline, genotypic testing revealed resistance to ganciclovir, foscarnet, cidofovir or letermovir in 55.1% of patients receiving maribavir (Supplementary Table 1). No baseline maribavir resistance was detected. CMV disease was present in 17 patients (34.7%) at the time of switch to maribavir as summarized in Table 1. Five LTx recipients (10.2%) had multiple CMV end-organ manifestations involving more than one organ system. All patients were at least once admitted to our hospital for i.v. ganciclovir ± CMVIgG or letermovir before rescue therapy with maribavir was initiated. Detailed information about detected anti-CMV resistance mutations and all medical interventions 6 months prior to maribavir is provided in the supplement material (Supplementary Table 1). Three months prior to initiation of maribavir, three patients were treated with rituximab due to the presence of donor-specific HLA antibodies, and two patients received eculizumab for atypical hemolytic uremic syndrome (aHUS). The proportion of patients experiencing myelosuppression (leukocytopenia/neutropenia) at initiation of maribavir therapy was 35.4% and 20.8%, respectively. 7 patients (14.3%) suffered from end-stage renal disease requiring dialysis. Demographics and characteristics of the study population are shown in Table 1.

**Table 1:** Demographics and characteristics of the study cohort

	All (n = 49)
Age (years)	60.2 ± 8.7
Sex (male), n (%)	24 (49.0)
BMI (kg/m^2^)	22.3 ± 3.6
Type of transplant, n (%)	
Bilateral lung transplantation (BLTx)	45 (91.8)
Single lung transplantation (SLTx)	4 (8.2)
Underlying diseases	
COPD, n (%)	14 (28.6)
ILD, n (%)	30 (61.2)
CF, n (%)	2 (4.1)
Others, n (%)	3 (6.1)
CMV-serostatus	
D + /R-	34 (69.4)
D + /R +	11 (22.4)
D−/R +	3 (6.1)
D−/ R-	1 (2.0)
Maintenance immunosuppression*, n (%)	
FK-506 + MMF + prednisone	40 (81.6)
CsA + MMF + prednisone	1 (2.0)
FK-506 + MMF + prednisone + mTOR**	8 (16.3)
CMV disease: organs, n (%)/patients, n (%)	25 (51.0)/17 (34.7)
CMV pneumonitis	7 (14.3)
CMV gastritis/esophagitis	6 (12.2)
CMV colitis	9 (18.4)
CMV retinitis	2 (4.1)
CMV encephalitis	1 (2.0)
End-stage renal disease requiring dialysis, n (%)	7 (14.3)

Data are presented as mean ± standard deviation and number and percentage, respectively

*BLTx* bilateral lung transplantation, *BMI* body mass index, *CF* cystic fibrosis, *CMV* cytomegalovirus, *COPD* chronic obstructive pulmonary disease, *CsA* ciclosporin A, *FK-506* tacrolimus, *ILD* interstitial lung diseases, *LTx* lung transplantation, *MMF* mycophenolate mofetil

*Immunosuppression before initiation of rescue therapy, **Everolimus or Sirolimus

### Maribavir, concomitant therapy & CMV-specific cell-mediated immunity

In most of the patients (79.6%) monotherapy with maribavir was initiated and no further action was taken. In 10 LTx recipients (20.4%) one or more additional antiviral interventions were performed simultaneously (+ 2 months) to initiation of maribavir. Simultaneous interventions were as follows: application of letermovir (n = 1), application of CMVIgG (n = 6), application of foscarnet, CMVIgG, letermovir and CMV-specific T-cells (n = 1), application of letermovir and CMV-specific T-cells (n = 1) and switch to mTOR-inhibitor-based quadruple low tacrolimus immunosuppression regimen as well as application of and CMVIgG (n = 1).

CMV-specific T-cells directed to CMV-IE1 and CMV-pp65 were measured by interferon-γ ELISPOT assay at a median of 55.5 days [range: 2–160] after initiation of therapy with maribavir. In total 45 tests have been performed in the presented cohort as summarized in Table 2. 16 tests have been performed before initiation of maribavir and 3 demonstrated a positive result despite having uncontrolled CMV infection. During therapy with maribavir 29 tests have been performed and 12 tests were positive. Thereof, 3 were already positive before initiation of maribavir, 8 have been tested before initiation of maribavir and were negative. Time from initiation of maribavir and assessment of CMV-specific T-cell-immunity was 58 [7–153] and 41 [2–149] days in patients with negative and positive results, respectively. Time from initiation of maribavir and assessment of CMV-specific T-cell-immunity was 49.5 [7–149] and 75 [2–160] days in responder and non-responder, respectively.

**Table 2:** Characteristics of the study patients after initiation of maribavir

	All (n = 49)	Responder (n = 34)	Non-responder (n = 15)	p-value
Age (years)	60.2 ± 8.7	59.7 ± 9.8	61.6 ± 4.8	0.484
Sex (male), n (%)	24 (49.0)	15 (44.1)	9 (60.0)	0.364
BMI (kg/m^2^)	22.3 ± 3.6	21.9 ± 3.3	23.1 ± 4.3	0.335
Number of CMV-reactivations before rescue therapy	2 (1–7)	3 (1–7)	2 (1–5)	0.108
Time until maribavir treatment after LTx (months)	15.5 (1.7–132.4)	17.9 (3.3–134.1)	13.8 (1.7–58.6)	0.178
CMV viral load (IU/ml) at initiation of maribavir	2.790 (81–1.600.000)	2.515 (81–93.600)	10.200 (91–1.600.000)	0.066
CMV viral load > 10.000 IU/ml at initiation of maribavir, n (%)	15 (30.6)	7 (20.6)	8 (53.3)	**0.047**
Duration of therapy with maribavir (days)*	82 (10–407)	75 (10–207)	122 (49–407)	0.001
Number of repeat treatments**	1.5 ± 0.7	1.6 ± 0.7	1.2 ± 0.4	0.065
Presence of CMV organ disease, n (%)	17 (34.7)	8 (23.5)	9 (60.0)	**0.028**
CMV-specific cell-mediated immunity before MAR, n positive/performed tests (%)***	3/16 (18.8)	2/11 (18.0)	1/5 (20.0)	1.000
CMV- specific T- cells (spot/2.0 × 10^5^ cells) before MAR				
IE-1	1.2 ± 3.2	1.6 ± 4.0	0.7 ± 0.8	0.611
pp65-antigen	10.4 ± 28.1	14.5 ± 35.3	3.5 ± 5.2	0.467
CMV-specific cell-mediated immunity during/after MAR, n positive /performed tests (%)****	12/29 (41.4)	11/20 (55.0)	1/9 (11.1)	**0.022**
CMV- specific T- cells (spot/2.0 × 10^5^ cells) during/after MAR				
IE-1	5.2 ± 8.5	6.8 ± 9.5	1.1 ± 3.2	**0.026**
pp65-antigen	18.7 ± 34.8	25.8 ± 39.2	0.9 ± 1.7	**0.010**
Maribavir resistance, n (%)	14 (28.6)	4 (11.8)	10 (66.7)	** < 0.001**
1st round	10 (20.4)	1 (2.9)	9 (60.0)	
2nd round	3 (6.1)	2 (5.9)	1 (6.7)	
3rd round	1 (2.0)	1 (2.9)	0 (0.0)	
Antiviral monotherapy with maribavir, n (%)	39 (79.6)	31 (91.2)	8 (53.3)	**0.005**
Time to decrease of 1 log_10_ after initiation of maribavir (days)^#^	11 (2–85)	10.5 (2–27)	14 (6–85)	**0.033**

Data are presented as mean ± standard deviation and number and percentage, respectively. CMV-specific T-cells directed to CMV- IE1 and CMV- pp65 were measured by interferon- γ Elispot assay

*BMI* body mass index, *CMV* cytomegalovirus, *CMV-CMI* CMV-specific cell-mediated immunity, *MAR* maribavir

*Cumulative time in the first round of MAR; three patients are still on therapy with maribavir.**13 patients received two treatment courses, and 5 patients underwent three courses. ***16 CMV-specific immunity tests were available before initiation of maribavir, performed in 13 patients. ****29 CMV-specific immunity tests were available during or after maribavir treatment, performed in 27 patients. ^#^available in 11 of 15 non-responder

### Efficacy, safety of maribavir & resistance

**Fig. 1:**
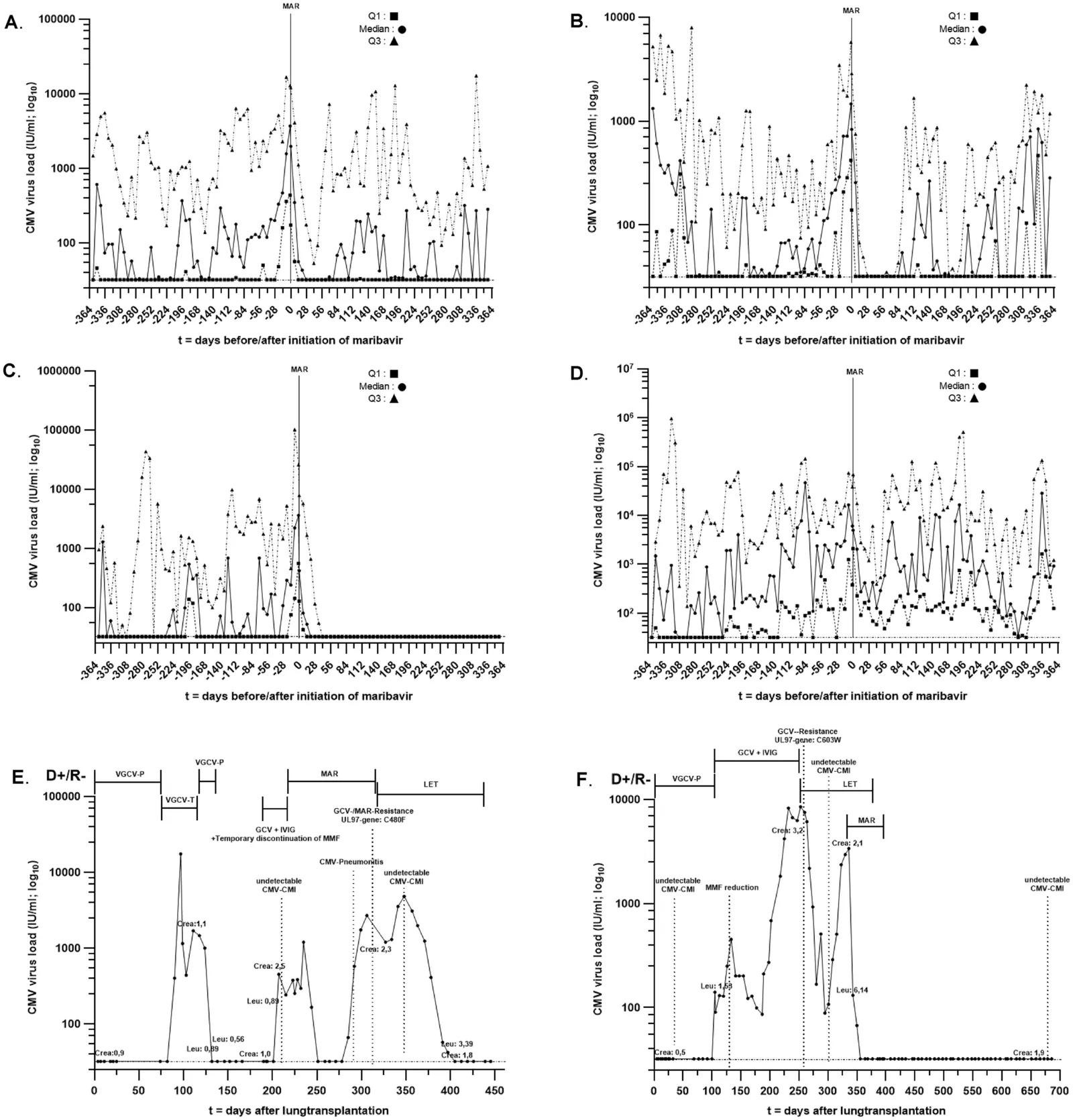

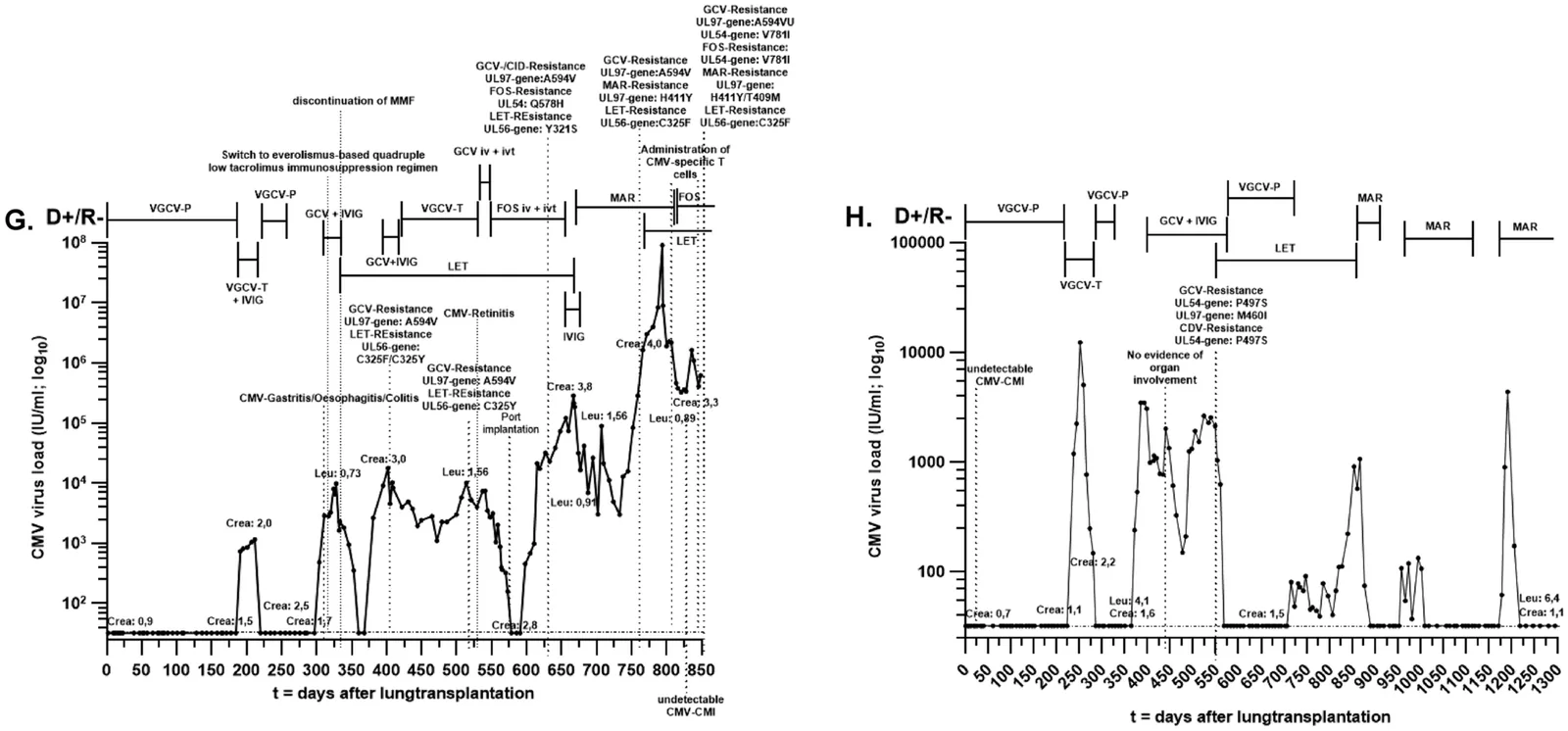
Course of CMV viral load before and after initiation of maribavir (**A**–**D**): **A** all patients (n = 49); **B** responders with relapse (n = 16); C) responder without relapse (n = 17); **D** all non-responders (n = 15); Course of CMV viral load after transplantation (**E**–**H**): **E** successful switch to letermovir; F) successful combination therapy of maribavir and letermovir; **G** Treatment failure after multiple interventions. **H** Successful treatment of CMV relapses. Data are provided as mean and quartile 1 and 3 (A-D) and as exact individual viral load trajectories (**E**–**H**). The horizontal dashed line indicates the threshold for quantitative detection of CMV plasma DNA by the assays used in this series (32 IU/ml)

Rescue therapy with maribavir was initiated at a median of 16.4 [1.7–134.1] months after LTx and was administered for a median duration of 82 [10–407] days in the first round, 95 [14–415] days in the second round, and 140 [81–198] days in the third round, respectively. The course of CMV viral load across different subgroups before and after initiation of maribavir is depicted in Fig. 1A–D. 34 patients (69.4%) were classified as treatment responder. CMV viral load could be significantly (> 1 log) decreased after 14 days in 29 patients (85.2%) and cleared subsequently in all patients after a median of 24.8 [7–54] days. LTx recipients with a negative treatment response had a significantly higher proportion of viral loads > 10.000 IU/ml at the initiation of maribavir (53.3% vs. 20.6%, p = 0.047) and longer time to decrease of 1 log_10_ after initiation of maribavir (14 (6–85) vs. 10.5 (2–27) days, p = 0.039) (Table 2). In five patients, treatment was switched to letermovir during the further course due to lack of response (Fig. 1 E). One patient was successfully treated with an initial combination therapy of maribavir and letermovir (Fig. 1F), whereas two patients showed no treatment response despite simultaneous therapy with maribavir and letermovir, due to various clinical factors (Fig. 1G).

In responders, maribavir was stopped at a median of 50 [5–183] days after the first negative CMV- PCR. Thereof, 16 patients (47.1%), in whom maribavir had been stopped due to a rapid response 58.5 [5–139] days after the last negative PCR or treatment-emergent adverse events (TEAEs) (n = 1), experienced relapse of CMV. One relapse of CMV was observed while the patient was on rescue therapy. Seven relapses of CMV occurred within 90 days after the first negative CMV PCR. 14 out of 16 patients (87.5%) were successfully retreated with maribavir. Five patients experienced a third relapse of CMV after successful prior therapies (Fig. 1H). In 14 of 16 (87.5%) patients with CMV relapse no modification of the immunosuppressive regimen was performed.

In case of suspicion of treatment failure resistance testing was performed in 18 patients on request of the treating physician. Thereof, 14 tests revealed emergence of maribavir resistance at a median of 132 [62–508] days of rescue therapy (Supplementary Table 2). Ten resistance mutations were first detected during the first course of maribavir, three during the second, and one during the third course of treatment. Among the 15 non-responders, three patients achieved complete CMV DNA clearance after switching to letermovir. Three additional patients were switched to letermovir and subsequently maintained persistent low-level CMV viral load (approximately 100–500 IU/mL) without evidence of disease progression. Two patients continued maribavir despite persistent low-level CMV viral load (approximately 200–500 IU/mL), as they had previously failed letermovir and harbored ganciclovir-resistant CMV. Both remained free of CMV end-organ disease and maintained stable allograft function. The remaining seven patients experienced progressive CMV viral load despite multiple salvage treatment strategies. Salvage therapies included ganciclovir plus letermovir (n = 2), foscarnet (n = 4), and a sequential combination regimen consisting of letermovir plus ganciclovir followed by foscarnet plus CMV hyperimmune globulin (Cytotect®) (n = 1). Despite these treatment modifications, none of these seven patients achieved CMV DNA clearance.

In multivariable logistic regression analysis, higher baseline CMV viral load was independently associated with maribavir resistance (OR 3.729 per log10 increase, 95% CI 1.360–10.226; p = 0.011). High-risk CMV serostatus (D + /R −) showed a non-significant trend toward increased odds of resistance (OR 1.530, 95% CI 0.950–2.360; p = 0.071), whereas the number of previous CMV reactivations, CMV disease, and initial maribavir monotherapy were not associated with resistance development (Table 3).

**Table 3:** Multivariate logistic regression analysis (OR, CI) to identify risk factors associated with evolution of maribavir resistance

All patients (n = 49)	Maribavir resistance		
Variable	OR	95% CI	p-value
Number of CMV-reactivations before rescue therapy	1.299	0.788–2.142	0.305
CMV viral load (log_10_ (IU/mL)) at initiation of maribavir	**3.729**	**1.360–10.226**	**0.011**
Presence of CMV disease, n (%)	2.044	0.374–11.159	0.409
Initial monotherapy with maribavir, n (%)	3.073	0.500–18.879	0.225
High-risk CMV serostatus (D + /R–)	**1.530**	**0.950–2.360**	**0.071**

*CI* confidence interval, *CMV* cytomegalovirus, *OR* odds ratio

Baseline CMV viral load was entered as log_10_ (IU/mL); the OR therefore represents the effect of a tenfold increase in CMV viral load. Continuous variables were entered per one-unit increase (number of previous CMV reactivations and log_10_ CMV viral load). Binary variables were coded as yes versus no

TEAEs occurred in 19 patients (38.8%), were mild and of gastrointestinal nature: diarrhea (n = 9, 18.4%), loss of appetite (n = 9, 18.4%), nausea/vomiting (n = 8, 16.3%), abdominal pain (n = 6, 12.2%), dysgeusia (n = 8, 16.3%), fatigue (n = 2, 4.1%), headache (n = 3, 6.1%), dizziness (n = 2, 4.1%), arthralgia (n = 1, 2.0%); anemia (n = 4, 8.2%) and peripheral edema (n = 1, 2.0%). Nephrotoxic events and myelosuppression did not occur or were not aggravated, respectively. In four cases TEAEs (n = 1, anemia and n = 3, gastrointestinal TEAEs) led to premature discontinuation of therapy. In one case, a dose reduction was performed due to TEAEs, and a relapse of CMV occurred during the course.

## Discussion

Antiviral agents are the mainstay for the treatment of CMV infection and disease, particularly in immunocompromised individuals such as solid organ transplant recipients [4]. Although R/R CMV occurs generally relatively rarely, its management remains challenging and is associated with substantial costs, morbidity and mortality [18]. Our findings highlight the therapeutic potential of maribavir in managing R/R CMV infections in a real world setting at large transplant center. Furthermore, maribavir was safe and well tolerated. Finally, high relapse rates and the emergence of resistance pose a significant challenge.

In small number of cases (20.4%) maribavir was used in combination with other antiviral interventions, like administration of CMVIgG or second antiviral drugs and adjustment of immunosuppressive therapy. Therefore, the beneficial effect on CMV viral load cannot be solely attributed to maribavir and has to be regarded in the context of several previous and simultaneous antiviral measures. However, the present findings extend and complement the pivotal phase III SOLSTICE trial, which established maribavir as an effective therapeutic option in the R/R CMV setting [6]. Although our response rate was numerically higher than that reported in the SOLSTICE lung transplant subgroup, direct comparisons should be interpreted cautiously because of differences in study design and patient characteristics [6]. This difference may also be attributed to the fact that a greater proportion of patients in the clinical study had an intermediate or high viral load at the initiation of rescue therapy. Notably, the time to significant decrease of viral load (i.e., ≥ 1 log10) and subsequent viral clearance were relatively short, highlighting maribavir’s rapid action in controlling CMV replication. Therefore, a 2-week period appears appropriate to assess the initial virological response to maribavir therapy, whereas longer follow-up is required to determine eventual CMV DNA clearance [18]. While the phase III SOLSTICE trial stratified patients according to predefined baseline CMV viral load categories (< 9,100, 9,100– < 91,000, and ≥ 91,000 IU/mL), emerging evidence suggests that viral loads > 10,000 IU/mL may already be associated with an increased risk of treatment failure and resistance emergence. Accordingly, a threshold of 10.000 IU/mL was used in the present study [6, 20].

However, the relapse rate observed in 47.1% of the responders after discontinuation of maribavir raises important questions regarding the optimal duration of therapy. Treatment duration was varying in our study, which was based on clinical grounds (e.g. TEAEs, positive CMV-specific immunity or viral clearance). In the majority of the patients (87.5%), rechallenge with maribavir was successful. Reduction of immunosuppression, whenever clinically feasible, represents an important component of CMV management in transplant recipients. Continued immunosuppression has been proposed as a major contributor to CMV recurrence following treatment cessation. In line with the data from the SOLSTICE study, where approximately 50% of patients developed recurrent CMV DNAemia after maribavir treatment, we likewise observed a high relapse rate among patients whose immunosuppressive regimen remained unchanged [6]. However, reducing immunosuppression is often difficult in lung transplant recipients due to the considerable risk of acute rejection and the development or progression of chronic lung allograft dysfunction (CLAD).

Furthermore, prolonged exposure to antiviral drugs may promote the development of viral resistance [18]. The detection of letermovir and maribavir resistance mutations highlights an ongoing clinical challenge in the management of CMV [12, 13, 21]. Maribavir resistance mutations most often occur in the UL97 gene and resistance-associated variants (RAVs) [13, 21]. In our study cohort, typical mutations such as T409M, H411Y, C480F have all been detected in several repeats of treatments [22]. Although many maribavir resistance mutations do not substantially affect susceptibility to ganciclovir, selected UL97 mutations, including C480F and F342Y, may confer cross-resistance or reduced susceptibility to both maribavir and ganciclovir. Consequently, genotypic resistance testing is important when selecting salvage treatment after maribavir failure [23]. In line with previous reports, maribavir resistance mutations were detected in 66.7% of maribavir non-responders [13, 22]. The most significant risk factor for resistant CMV across multiple trials is the lack of prior CMV immunity, particularly in CMV mismatched D + /R − recipients [24]. In our cohort, higher baseline CMV viral load was independently associated with the development of maribavir resistance, whereas CMV-mismatched D + /R − serostatus showed a non-significant trend toward an increased risk. These findings suggest that a high viral burden at treatment initiation may facilitate the emergence of resistance and underscore the importance of early antiviral intervention before substantial viral replication has occurred. CMV-specific T-cell immunity plays a central role in host defense after lung transplantation, and several studies have demonstrated its association with CMV control and transplant outcomes. While its routine use in clinical decision-making is not yet supported by sufficient evidence, CMV-specific immune monitoring remains a promising tool for future individualized CMV management and warrants further prospective evaluation [25–27]. However, CMV-specific T-cell assays are not yet routinely implemented in clinical practice in many transplant centers.

Reported treatment-emergent adverse events were mild and mostly of gastrointestinal nature (e.g., loss of appetite, nausea, vomiting, and diarrhea). Nephrotoxic events did not occur. Other currently available antiviral drugs, such as foscarnet and cidofovir, are linked to substantial metabolic alterations and nephrotoxicity. A dose reduction due to adverse events may lead to a relapse of CMV infection and therefore requires close monitoring. Premature discontinuation due to adverse events occurred in only a minority of cases. Both letermovir and maribavir have favorable safety profiles compared to CMV DNA polymerase inhibitors, without the risk of neutropenia and leukopenia associated with ganciclovir and renal toxicities associated with foscarnet and cidofovir. Moreover, letermovir and maribavir are orally bioavailable, which allows convenient outpatient treatment [4, 6, 11].

The results of our study should be interpreted in light of the study design and its inherent limitations, including its retrospective, single-center nature and the absence of a control group. Furthermore, because baseline genotypic testing for maribavir resistance was not routinely available, we cannot exclude that some UL97 resistance mutations detected during treatment were already present before initiation of maribavir therapy. Furthermore, the threshold for initiating maribavir therapy in difficult-to-treat CMV infection has evolved over time. Initially, maribavir was primarily used after obtaining cost coverage approval and following failure of salvage therapies such as foscarnet or letermovir. Over the course of the study period, however, maribavir was increasingly introduced earlier in the treatment algorithm and eventually became the preferred first-line therapy for resistant or refractory CMV infection. In addition, the cohort was characterized by considerable heterogeneity in CMV management strategies, reflecting changes in clinical practice and varying treatment approaches over time. Furthermore, approximately half of the patients had received letermovir prior to maribavir, representing a challenging and somewhat unique clinical scenario, as the use of letermovir for treatment of CMV infection is not widely adopted and is currently not recommended by clinical guidelines. The number of patients receiving combination therapy in this study was too low to accurately describe the combined effect. While this heterogeneity mirrors real-world clinical care and enhances the generalizability of our findings, it complicates the interpretation of treatment outcomes and limits the ability to draw definitive conclusions regarding the efficacy and optimal use of maribavir in this setting.

In conclusion, maribavir represents an important therapeutic option for the treatment of R/R CMV infection after lung transplantation, particularly given its favorable safety profile and the lack of nephrotoxicity or myelosuppression. However, treatment failure, relapse after treatment discontinuation, and the detection of UL97 resistance mutations during therapy remained common in this cohort, highlighting the need for careful virological monitoring and resistance testing in patients with persistent or recurrent CMV DNAemia. Further studies are warranted to define the optimal treatment duration and strategies to prevent relapse and resistance.

## Supplementary Information

Below is the link to the electronic supplementary material.

Supplementary file1 (DOCX 23 KB)

## Data Availability

Data are available on request from the authors.

## Abbreviations

aHUS: Atypical hemolytic uremic syndrome
ANC: Absolute neutrophil count
CDV: Cidofovir
CMV: Cytomegalovirus
CMVIgG: I.v. CMV-immune globulins
CsA: Ciclosporin A
FK-506: Tacrolimus
FOS: Foscarnet
GCV: Ganciclovir
G/L: Giga per liter (10⁹/L)
HSCT: Hematopoietic stem cell transplant
LTx: Lung transplantation
MAR: Maribavir
MMF: Mycophenolate mofetil
mTOR: Mammalian target of rapamycin
PCR: Polymerase chain reaction
R/R: Resistant or refractory
SOT: Solid organ transplantation
TEAEs: Treatment-emergent adverse events
