# Supplementary material for: Maribavir in lung transplant recipients with cytomegalovirus infection: real-life experience
Source: Infection. 2026 Aug 18;54(5):2797–807. doi: 10.1007/s15010-026-02927-3 (PMC13727892; doi:10.1007/s15010-026-02927-3)
Supplement: Supplementary file 1 [file 15010_2026_2927_MOESM1_ESM.docx]

**Supplementary Material**

**Maribavir in lung transplant recipients with cytomegalovirus infection: real-life experience**

Tobias **Veit,** Stefan **Trost,** Teresa **Kauke,** Nina **Engels,** Gabriela **Leuschner,** Michael **Gerckens,** Carlo **Mümmler,** Sebastian **Michel,** Michael **Zoller,** Valeria **Hackemann,** Jürgen **Behr,** Nikolaus **Kneidinger**

**Table S1.** Therapeutic interventions during the 6 months before initiation of maribavir.

|  | **All**  **(n=49)** | **Responder**  **(n=34)** | **Non-Responder**  **(n=15)** | **p-value** |
| --- | --- | --- | --- | --- |
| Age (years) | 60.2 ± 8.7 | 59.7 ± 9.8 | 61.6 ± 4.8 | 0.484 |
| Sex (male), n (%) | 24 (49.0) | 15 (44.1) | 9 (60.0) | 0.364 |
| Time to first CMV-infection after LTx (weeks) | 5.0 (0.9-48.0) | 11.8 (0.9-44.0) | (2.4-48.0) | 0.601 |
| Number of CMV-reactivations before rescue therapy | 2 (1-7) | 3 (1-7) | 2 (1-5) | 0.108 |
| Ganciclovir-resistance, n (%)^*^ | 19 (38.8) | 10 (29.4) | 9 (60.0) | 0.729 |
| UL 97 Gene Mutation, n (%) | 11 (22.4) | 5 (14.7) | 6 (40.0) | 0.095 |
| UL 54 Gene Mutation, n (%) | 2 (4.1) | 0 (0.0) | 2 (13.3) | 0.095 |
| UL 97 + UL 54 Gene mutation, n (%) | 6 (12.2) | 5 (14.7) | 1 (6.7) | 0.630 |
| Cidofovir-resistance, n (%)* | 9 (18.4) | 7 (20.6) | 2 (9.1) | 0.692 |
| Foscarnet-resistance, n (%)* | 1 (2.0) | 0 (0.0) | 1 (6.7) | 0.218 |
| Letermovir-resistance, n (%)* | 17 (34.7) | 7 (20.6) | 10 (66.7) | **0.004** |
| Use of CMV-immune globulins, n (%)  Episodes of CMV-immune globulins use** | 20 (40.8)  0.6 ± 0.8 | 11 (32.4)  0.3 ± 0.5 | 9 (60.0)  1.1 ± 1.2 | 0114  **0.001** |
| Use of (val)ganciclovir, n (%)  Cumulative duration of (val)ganciclovir in therapeutic dose  (days) | 43 (87.8)  66.5 ± 48.0 | 27 (79.4)  59.3 ± 47.2 | 15 (100.0)  80.0 ± 48.1 | 0.093 |
| Change in management of MMF, n (%)  Dose reduction, n (%)  Discontinuation of MMF, n (%) | 32 (65.3)  16 (32.7)  16 (32.7) | 22 (64.7)  13 (38.2)  9 (26.5) | 10 (66.7)  3 (20.0)  7 (46.7) | 1.000 |
| Use of foscarnet, n (%)  Duration of foscarnet therapy (days) | 3 (6.1)  (5-127) | 1 (2.0)  (5) | 2 (13.3)  (16-127) | 0.218 |
| Use of letermovir, n (%) | 22 (44.9) | 13 (38.2) | 9 (60.0) | 0.217 |
| Switch to mammalian target of rapamycin (mTOR)-inhibitor-based quadruple low tacrolimus immunosuppression regimen, n (%) | 3 (6.1) | 1 (2.0) | 2 (13.3) | 0.235 |

Data are presented as mean ± standard deviation and number and percentage or median and IQR, respectively. Definition of abbreviations: CMV, cytomegalovirus; LTx, lung transplantation; MMF, mycophenolate mofetil. ^*^Tested in 33/49 patients. **CMV-immune globulins (CMVIgG; Cytotect®) with a dose of 100 IE (Ehrlich units)/kg/day (= 1ml/kg/day and 50mg/kg/day, resp.) for 5-7 days.

**Table S2.** UL56-, UL97- and UL54-gene mutations and published ganciclovir ratios indicating the degree of resistance in patients with maribavir resistance.

| **Patient ID** | **UL56-gene** | **UL97-gene** | **UL54-gene** | **Published**  **GCV-ratios^1-6^** | **CDV or FOS**  **resistance** |
| --- | --- | --- | --- | --- | --- |
| 1 | C325F | H411Y^M^ | - |  | no / no |
| 2 | C325F, Y321S | A594V^G^, H411Y^M^ | Q578H | 8,3 / 3.3 | yes / yes |
| 3 | C325Y | T409M^M^ | T504I^G, C^ |  | yes / no |
| 4 | R369S | T409M^M^ | - |  | no / no |
| 5 | C325F | L592F^G^, T409M^M^ | - | 15.7 | no / no |
| 6 | C325Y | C480F^M, G^, M460V^G^ | - | 8.3 | no / no |
| 7 | - | H411Y^M^ | - |  | no / no |
| 8 | - | C480F^M, G^ | - |  | no / no |
| 9 | - | T409M^M^ | - |  | no / no |
| 10 | - | H411Y^M^, L595S ^G^ |  | 9.2 | no / no |
| 11 | - | T409M^M^ | - |  | no / no |
| 12 | - | T409M^M^ | - |  | no / no |
| 13 | - | C480F^M, G^ | - |  | no / no |
| 14 | - | T409M^M^, C480F^M, G^ | - |  | no / no |

Definition of abbreviations: CDV, cidofovir; FOS, foscarnet; GCV, ganciclovir. Using sequencing of sections of UL97-gene (Codon 312-666), UL54-gene (Codon 265-1053), UL56-gene (Codon 12-393). GCV ratio = inhibitory concentration IC_50_ of mutant/IC_50_ of wild-type. ^G^mutation associated with GCV resistance, ^m^mutation associated with maribavir resistance.

References:

1) El Chaer, Firas et al. “How I treat resistant cytomegalovirus infection in hematopoietic cell transplantation recipients.” Blood vol. 128,23 (2016): 2624-2636.

doi:10.1182/blood-2016-06-688432

2) Göhring K, Hamprecht K, Jahn G. Antiviral Drug- and Multidrug Resistance in Cytomegalovirus Infected SCT Patients. Comput Struct Biotechnol J.

2015;13:153-159. Published 2015 Feb 10. doi:10.1016/j.csbj.2015.01.003

3) Lurain NS, Chou S. Antiviral drug resistance of human cytomegalovirus. Clin Microbiol Rev. 2010 Oct;23(4):689-712. doi: 10.1128/CMR.00009-10.

4) Chou S. Rapid In Vitro Evolution of Human Cytomegalovirus UL56 Mutations That Confer Letermovir Resistance. Antimicrob Agents Chemother. 2015

Oct;59(10):6588-93. doi: 10.1128/AAC.01623-15. Epub 2015 Aug 10. PMID: 26259791; PMCID: PMC4576131.

5) Zhu VZ, Horton MB, Haeusler GM, Yong MK. The emergence of letermovir and maribavir drug-resistant mutations: from clinical trials to real-world studies.

Curr Opin Infect Dis. 2024 Dec 1;37(6):536-546. doi: 10.1097/QCO.0000000000001065. Epub 2024 Sep 27. PMID: 39331647.
